# Characterization of Aliphatic Polyesters Synthesized via Enzymatic Ring-Opening Polymerization in Ionic Liquids

**DOI:** 10.3390/molecules22060923

**Published:** 2017-06-02

**Authors:** Urszula Piotrowska, Marcin Sobczak, Ewa Oledzka

**Affiliations:** 1 Department of Biomaterials Chemistry, Chair of Inorganic and Analytical Chemistry, Faculty of Pharmacy with the Laboratory Medicine Division, Medical University of Warsaw, 1 Banacha Street, Warsaw 02-097, Poland; marcin.sobczak@wp.pl (M.S.); eoledzka@wum.edu.pl (E.O.); 2 Department of Organic Chemistry and Biochemistry, Faculty of Materials Science and Design, Kazimierz Pulaski University of Technology and Humanities in Radom, 27 Chrobrego Street, Radom 26-600, Poland

**Keywords:** aliphatic polyesters, biomedical polymers, drug delivery systems, ionic liquids, lipases, polylactide, ε-caprolactone, ring-opening polymerization

## Abstract

To evaluate the effects of ionic liquids (ILs) on the microstructural features of aliphatic polyesters for biomedical applications, a series of copolymers were synthesized by lipase ring opening polymerization of *rac*-lactide (*rac*-LA) and ε-caprolactone (CL). The chemical structures of resulting polymers were characterized by ^1^H- and ^13^C-NMR and the average molecular weight (*M_n_*) and dispersity index were characterized by gel permeation chromatography. The structure of the copolymers confirms the presence of linear polymer chains with end-functional hydroxyl groups allowing covalent coupling of the therapeutic agents. Chain microstructure of copolymers indicates the presence of both random and block copolymers depending on the synthesis conditions. Moreover, it was found that CL is the most active co-monomer during copolymerization which enhances the polymerizability of *rac*-LA and allows to obtain higher *M_n_* of the copolymers. The results demonstrate that ILs could be promising solvents in synthesis of aliphatic esters for biomedical applications.

## 1. Introduction

Poly(ε-caprolactone) (PCL) and polylactide (PLA) are two of the most commonly used polyesters for biomedical applications due to their good biocompatibility and lack of toxicity [1,2]. These polymers are very attractive because they degrade in vivo by hydrolytic deesterification during the carboxylic acid cycle and are subsequently excreted as carbon dioxide and water. PCL is known to be a semicrystalline, linear, aliphatic polyester with a slow degradation rate [3]. In the case of PLA, it is possible to obtain polymers with various crystallinities depending on the type of monomer used in the synthesis of polymer. PLA can be obtained by polycondensation from lactic acid or by ring-opening polymerization (ROP) of a lactide (LA) which is a cyclic dimer of lactic acid. LA monomers exist in three different stereoisomer forms: d-lactide (d-LA), l-lactide (l-LA) and *meso* d,l-lactide (d,l-LA). A racemic mixture of d- and l-lactide is referred to as *rac*-lactide (*rac*-LA) [4]. Due to the significantly different properties (and thus rate of degradation) of PCL and PLA, biomaterials based on the LA and ε-caprolactone (CL) copolymers have attracted more attention in the fields of surgery, drug delivery systems and tissue engineering [5]. Improved copolymer properties strongly depend both on chemical composition and the chain microstructure.

ROP is the most common method of synthesizing polyesters for biomedical applications due to ability to control polymer properties and architecture during this process. The ROP of a cyclic ester can be performed in the presence of cationic/anionic initiators, metal coordinate and enzymatic catalysts [6]. Enzyme catalysis has provided an environmentally friendly, synthetic strategy for a variety of polyester-based biomaterials [7,8,9]. Several lipases have been tested as catalysts for the ROP of different polyesters [10,11,12], among which *Candia antarctica* lipase B (CALB) is the most promising biocatalyst. The enantioselectivity of the CALB is referred to as enabling a selective polymerization of d-LA and no polymerization of l-LA and its *meso* form [13,14]. Probably, the first monomer units during l-LA homopolymerization catalyzed by the immobilized form of CALB are opened. The resulting *S*-configuration of the secondary alcohol cannot undergo further monomer addition and causes the inhibition of the reaction. In contrast, *R*-configurated secondary alcohol obtained from d-LA is able to react with another enzyme activated monomer and thus to propagate the chain [15]. Düşkünkorur et al. [16] reported that CALB immobilized on acrylic resin is more active for the ROP of d-LA than for l-LA when reaction was conducted in toluene.

Interestingly, CALB seems to catalyze the ROP of l-LA in ionic liquids (ILs). ILs are attracting more attention as non-aqueous, green solvents for biocatalysis polymer synthesis [17,18]. Enzymes in ILs may have higher activity, enantioselectivity, regioselectivity and stability than in conventional organic solvents [17]. Yoshizawa-Fujita et al. conducted the first ROP of l-LA in ILs using free form of CALB. The polymers were obtained with a number-average molecular weight (*M_n_*) 54,600 g/mol and 43,800 g/mol with respect to the 1-butyl-3-methylimidazolium tetrafluoroborate ([bmim][BF_4_]) and 1-butyl-3-methylimidazolium bis((trifluoromethyl)sulfonyl)imide ([bmim][NTf_2_]) at 110 °C after 24 h [19]. Chanfreau et al. [20] also obtained poly(l-lactide) (PLLA) with high *M_n_* (37,800 g/mol) in 1-hexyl-3-methylimidazolium hexafluorophosphate ([hmim][PF_6_]) at 90 °C using the immobilized form of CALB. On the other hand, Mena et al. [21] obtained a low molecular weight PLLA with *M_n_* = 581 g/mol after 11 days of polymerization at 65 °C using immobilized form of CALB and 1-butyl-3-methylimidazolium hexafluorophosphate ([bmim][PF_6_]) as a solvent.

In this article, we performed for the first time the enzymatic copolymerization of CL and *rac*-LA in ILs in the presence of enzyme catalytic system in ionic liquids. We applied the most common ILs for biocatalysis, the imidazolium-based ionic liquids [bmim][BF_4_] and [bmim][NTf_2_]. The microstructures of a series of obtained copolymers with various compositions were analyzed by means of ^1^H and ^13^C-NMR, and the *M_n_* and dispersity index (*M_w_*/*M_n_*) were characterized by gel permeation chromatography (GPC). The aim of this work was to obtain polyesters with well-known microstructures with respect to their applications as matrices for drug release systems.

## 2. Results and Discussion

### 2.1. Copolymerization of rac-LA and CL

Copolymers of *rac*-LA and CL were synthesized during the ROP process in two 1-butyl-3-methylimidazolium cation-based ILs with two different anions: tetrafluoroborate and bis(trifluoromethylsulfonyl)imide at 80 and 110 °C for 7 to 14 days at various molar ratios of *rac*-LA/CL. The reactions were carried out using 100 mg (10 wt. %) *Candida antarctica* lipase B (CALB) as catalyst. Water from impurities (free water from an enzyme and ionic liquids) acts as a co-initiator [22]. Thermogravimetric analysis revealed that there was about c.a. 1.0 wt. % of free water in CALB [12] and c.a. 0.1 wt. % [12] and c.a. 1.3 wt. % in [bmim][NTf_2_] and [bmim][BF_4_], respectively (Figure S1, Supplementary Materials). The effects of polymerization parameters on the chain microstructures of the synthesized polyesters were investigated.

**Figure 1:**
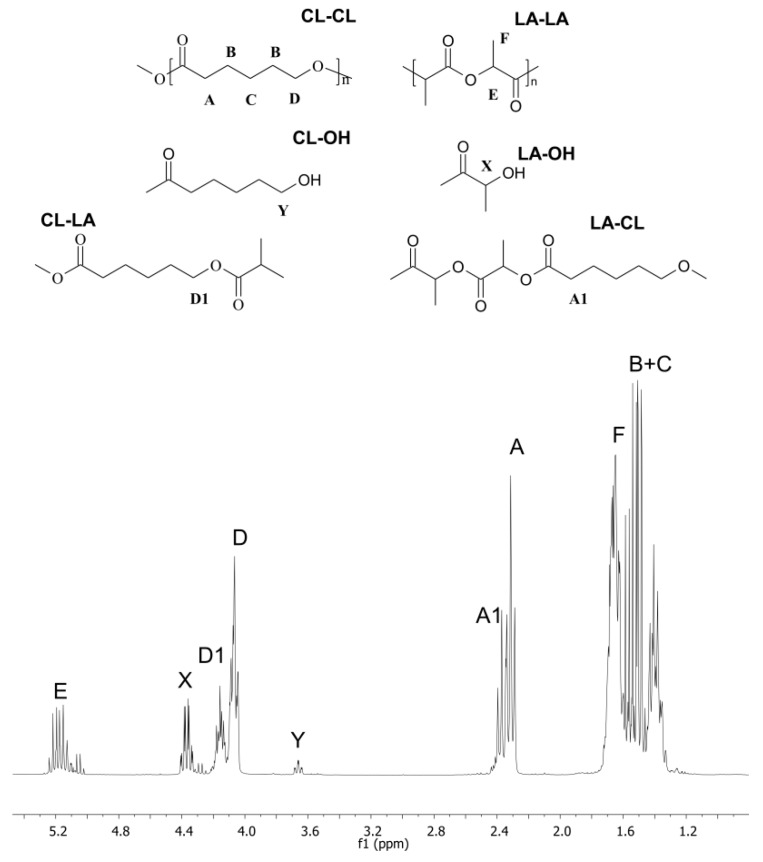
^1^H-NMR spectrum of copolymers of *rac*-LA and CL (entry A2).

The structure of the synthesized copolymers was analyzed by ^1^H-NMR and ^13^C-NMR. The ^1^H-NMR spectrum (CDCl_3_, 300 MHz) presented in Figure 1 confirmed the structure of the copolymers of *rac*-LA and CL.

The signals centered at 5.15 and 1.54 ppm were assigned to the lactide methine protons (CH, H_E_) and lactide methyl protons (CH_3_, H_F_). The signal at 4.35 ppm was assigned to the protons next to the end-functional hydroxyl groups of lactide (CH(CH_3_)OH, H_X_). Protons from heterodyads cause the signals at 4.16 ppm (CH_2_O, H_d1_) and at 2.39 ppm for (CH_2_, H_A1_). The signals at 4.07 (CH_2_O, H_d_) and 2.31 ppm (CH_2_CO, H_A_) correspond to the ε- and α-methylene protons of the PCL. Another feature is the chemical shift for protons from methylene groups of PCL next to the end-functional hydroxyl groups, which appear as a triplet at 3.66 ppm (CH_2_OH, H_Y_). Two other multiplets at c.a. 1.65 ppm and 1.40 ppm were attributed to the protons of the methylene groups of the PCL chain (CH_2_, H_B_, H_C_).

### 2.2. Chemical Composition of the Copolymers

**Figure 2:**
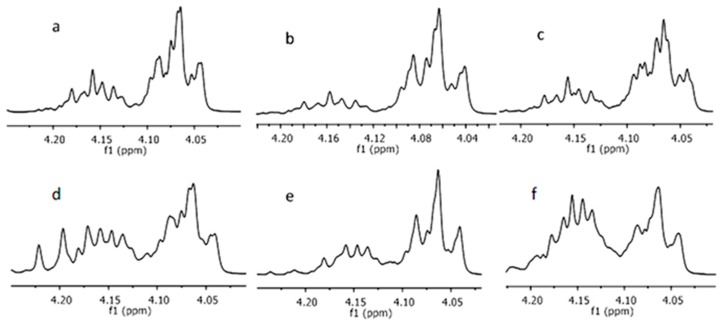
Expansion of the domain of interest in 1H-NMR spectra: (**a**) A2; (**b**) A5; (**c**) A8; (**d**) B2; (**e**) B5; (**f**) B8.

The chemical composition of copolymers was also calculated from the ^1^H-NMR spectra by taking the ratio of the peak areas corresponding to the *rac*-LA methine protons at δ = 5.1–5.3 ppm and the CL ε-methylene protons at δ = 4.0–4.1 ppm [23]. *rac*-LA conversion was determined by ^1^H-NMR in the comparison with integrated signal areas of the methylene groups next to the carbonyl group in the monomer at 5.08 ppm and the polymer at 5.15 ppm before the purification procedure. It should be noted that CL-LA heterodiads are overlapped in the LA methine signal and they have the same relative molar fraction as LA-CL dyad [23,24]. The results are summarized in Table 1 and presented in Figure 2.

As it can be seen in Table 1, in a case of [bmim][BF_4_] as a solvent, the incorporation of LA units into polymer chains was incomplete at each case even at higher temperature (110 °C) and generally remained at a similar, low level (0.25–0.42). During reactions in [bmim][BF_4_], color changes in the reaction medium were observed from yellow at 80 °C to dark brown at 110 °C. This demonstrates that tetrafluoroborate anion in ILs can degrade during long-term reactions in the presence of water (co-initiator), resulting in the formation of hydrogen fluoride which probably can cause an enzyme deactivation. Moreover, it should be emphasized here that [bmim][BF_4_] tend to absorb more water than [bmim][NTf_2_].

In a case of [bmim][NTf_2_] as a solvent, the copolymer composition was generally close to the feed molar ratio of monomers. For example, in the case of B8 the molar LA composition was 0.62 when 0.67 was expected from feed molar ratio. One exception is sample B7—the molar LA composition was only 0.36 in this case (0.67 was expected from the feed molar ratio), because of incomplete monomer consumption after 7 days of polymerization. This can be observed due to non-dissolved *rac*-LA in the reaction tube as a result of low co-monomer (CL) content and short reaction time. Moreover, we observed a color change in a solvent at 110 °C after 14 days. However, the change was less than that seen with copolymerization in [bmim][BF_4_].

These results are convergent with the results of the homopolymerization of *rac*-LA and CL (results not shown) and demonstrate that CL is the most active co-monomer in our experiments, which enhances the polymerizability of *rac*-LA. Moreover, CL allows higher conversion of *rac-*LA and allows a higher *M_n_* to be obtained for the copolymers [16]. For example, conversion of *rac*-LA synthesized in [bmim][NTf_2_] at 80 °C for 14 days increased from 0.59 (sample B8, CL molar ratio 0.38) to 0.86 (sample B2, CL molar ratio 0.54) and 0.94 (sample B5, CL feed molar ratio 0.67). For copolymers synthesized at 110 °C in [bmim][NTf_2_], *rac*-LA conversion remained at the same, high level, approximately 0.90 in each case.

The *M_n_* of copolymers of *rac*-LA and CL also depends on the *rac*-LA/CL molar ratio, the kind of solvent and their purity. The *M_n_* values of the copolymers determined by the GPC were in the range of 700–3100 g/mol and increased with increasing CL content in the feed. For example, *M_n_* of copolymers synthesized in [bmim][NTf_2_] at 110 °C for 14 days increased from 1700 g/mol (sample B9, *rac*-LA molar ratio 0.60) to 2700 g/mol (sample B3, *rac*-LA molar ratio 0.44) and 2900 g/mol (sample B6, *rac*-LA molar ratio 0.30). The results are consistent with the results reported by Wahlberg et al. for bulk copolymerization of CL with d,l-LA at 60–70 °C after 8 weeks [25].

The yield of the copolymerization process ranged from 52% to 69% for reaction in [bmim][BF_4_] and from 52% to 69% for reaction in [bmim][NTf_2_], and it was higher at higher temperatures.

### 2.3. Chain Microstructures of Synthesized Copolymers

**Figure 3:**
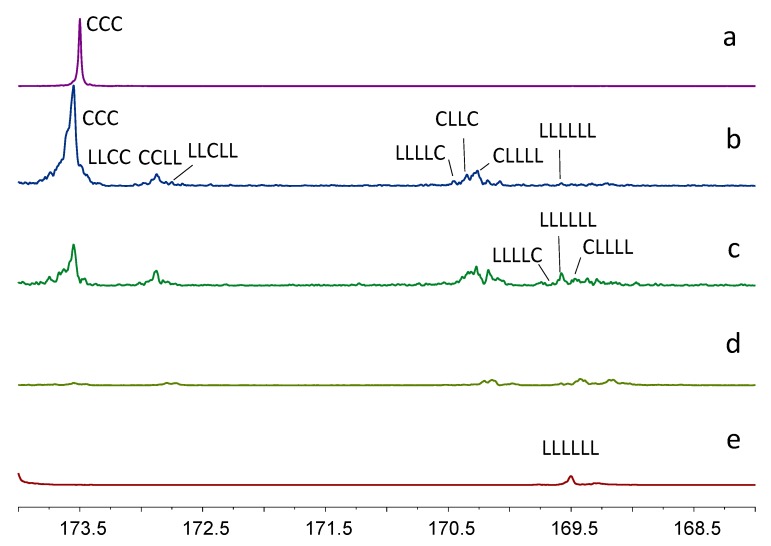
Expanded ^13^C-NMR spectra of carbonyl region of PCL, PLA and copolymers of PCL and PLA synthesized in [bmim][NTf_2_] at 110 °C for 14 days; (**a**) PCL; (**b**) B6; (**c**) B3; (**d**) B9; (**e**) PLA.

Microstructural analysis of the synthesized copolymers, both in terms of their monomer sequencing and average monomer block length, were conducted on the basis of the attribution of the peaks in the ^13^C-NMR spectra, since this technique is very sensitive to monomer sequencing. Moreover, it is a powerful tool for determining the average sequence length for each type of monomer unit [26]. Monomer sequencing was determined on the basis of the expanded carbonyl carbon signals from δ = 168–174 ppm at ^13^C-NMR spectra as previously described by Kricheldorf and Kreiser [27] and Kasperczyk and Bero [28]. We used various mixtures of co-monomers because reactivity ratios between these two monomers are quite different. Copolymers containing mainly lactidyl units are expected to be formed at an initial stage of a reaction because lactide monomer is generally preferentially incorporated in the polymer chain [25]. However, as the reaction proceeds, especially with long reaction times, a monomer sequencing tends to randomize. The process takes place as a result of transesterification reactions, which play an important role in the redistribution of monomer sequences and affect a polymer chain microstructure [24,29]. Kasperczyk and Bero for the first time defined two models of transesterification referred to as the first and the second modes. In the first transesterification mode, a cleavage of the polylactide block occurs between monomeric units and is connected to the appearance of a triad signals of CLLC. In the second mode of transesterification, an ester bond opening occurs between monomeric units as well as within lactidyl units [28]. Figure 3 shows the assignment of peaks in ^13^C-NMR spectra of the copolymers synthesized in [bmim][NTf_2_] at 110 °C.

The presence of the signals between 169.6–173.4 ppm indicates the occurrence of the intermolecular transesterification between caproyl (C) and lactydyl (LL) units of the polymer chains [30]. Since at the first reaction stage, mainly the first mode of transesterification proceeded, we recognized one transesterification type, referred to as the first mode. However, it is necessary to assume that the presence of the signal at 170.8 ppm, assigned as the carbonyl group of CLC triad (or CLLLC chain sequences at 170.3 ppm), resulted from the attack of active caproyl ends on the formed lactyl unit sequences (L), which could also indicate the presence of the second mode of the transesterification. However, by the end of the copolymerization, CLC triads were not detected, so we concluded that only the first mode of transesterification occurs at 110 °C. A similar effect was observed for [bmim][BF_4_] as a solvent (spectra not shown).

**Figure 4:**
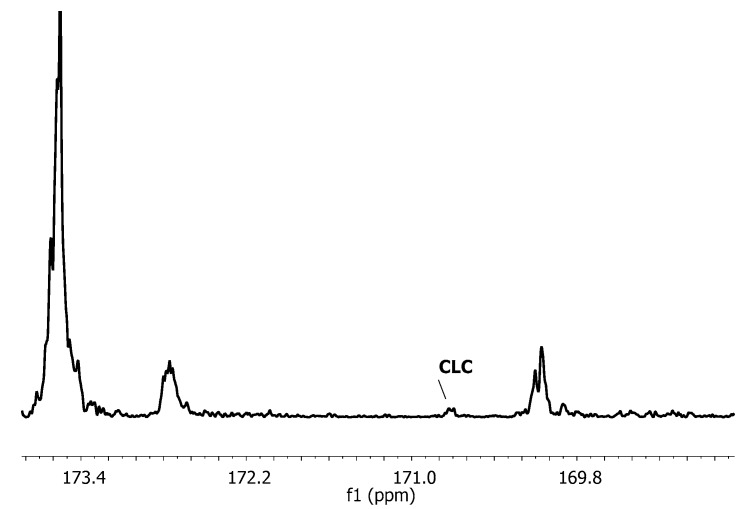
Expanded ^13^C-NMR spectra of carbonyl region of copolymer (B2) synthesized in [bmim][BF_4_] at 80 °C for 14 days.

Interestingly, in the analyzed microstructures of copolymers synthesized in ILs at 80 °C (samples A2, A5, B2, B5, with 0.50% and 0.67% CL in the feed), we observed low intensities of CLC sequences attributable to the second mode of transesterification (Figure 4).

It is well known from the ROP of lactones and lactides that the catalyst or the initiator causes transesterification reactions at high temperatures, or with long reaction times. Moreover, two different types of transesterification also compete with each other: (1) intermolecular transesterification between different polymer chains and (2) intramolecular transesterification (back-biting) within the same chain [31]. Probably, higher temperatures led to the formation of cyclic polymer chains at first, so intramolecular transesterification are dominating. Wahlberg et al. revealed that macrocyclic products were initially formed and gradually disappeared as the reaction proceeded [25]. In a case with a lower temperature, in the presence of the high feed ratio of a more active monomer, we recognized the existence of both the first and the second modes of transesterification. Moreover, the experimental results show that transesterification reactions are in minority in each case comparing with copolymerization.

Equations (1) and (2) were applied to obtain the number-average sequence lengths of the lactidyl and caproyl sequences [28,32]:

(1) $$l_{LL}^{e}=\frac{\left[\mathrm{LLLLLL}\right]+\left[\mathrm{LLLLC}\right]+\left[\mathrm{CLLLL}\right]+\left[\mathrm{CLLC}\right]}{\left[\mathrm{CLLC}\right]+\frac{1}{2}\left(\left[\mathrm{CLLLL}\right]+\left[\mathrm{LLLLC}\right]\right)}$$

(2) $$l_{Cap}^{e}=\frac{\left[\mathrm{LLCLL}\right]+\left[\mathrm{CCLL}\right]+\left[\mathrm{LLCC}\right]+\left[\mathrm{CCC}\right]}{\left[\mathrm{LLCLL}\right]+\frac{1}{2}\left(\left[\mathrm{CCLL}\right]+\left[\mathrm{LLCC}\right]\right)}$$

As it can be seen in Table 2, for copolymers synthesized in [bmim][NTf_2_], both $l_{LL}^{e}$ and $l_{Cap}^{e}$ values depend on the monomers feed ratio, and increased as the relative proportions of their respective monomer increased. In the case of [bmim][BF_4_] as a solvent, a stronger effect from the monomer feed ratio and temperature was observed for CL ($l_{Cap}^{e}$: 4.44–23.56) than for LA ($l_{LL}^{e}$: 1.23–2.87). Moreover, the higher $l_{Cap}^{e}$ values reflect the higher reactivity ratio of CL and longer but fewer C sequences than LL sequences within the copolymer chain. Shorter $l_{Cap}^{e}$ and $l_{LL}^{e}$ values indicate that transesterification indeed occurred.

A transesterification reaction rearranges the polymer sequences and leads to randomization. The degree of randomness (*R*) of the copolymer and the coefficient of the second mode of transesterification (T_II_) were calculated from the following equations [28,32]:

(3) $$R=l_{LL}^{R}/l_{LL}^{e}=l_{Cap}^{R}/l_{Cap}^{e}$$

where $l_{LL}^{e}$ and $l_{Cap}^{e}$ are the experimental average length of the lactidyl and caproyl blocks, and $l_{LL}^{R}\;$and $l_{Cap}^{R}$ are the average length of the lactidyl and caproyl blocks for the chains with random units distribution, as would be obtained by the complete transesterification via the first and the second mode, and it can be calculated from the following equations [28,32]:

(4) $$l_{LL}^{R}=\left(k+1\right)/2k$$

(5) $$l_{Cap}^{R}=\left(k+1\right)$$

(6) $$T_{\mathrm{II}}={\left[CLC\right]}_{e}/{\left[CLC\right]}_{R}$$

where [*CLC*]*_e_* is the experimentally determined concentration of *CLC* sequence from the ^13^C-NMR spectroscopy, and [*CLC*]*_R_* is the theoretical concentration for completely random chains calculated via Bernoullian statistics:

(7) $${\left[CLC\right]}_{R}=k^{2}/{\left(k+1\right)}^{3}$$

(8) k=[CL]/[rac-LA]

Considering that the *R* coefficient value equals 1 for completely randomness (and also amorphous copolymers) and 0 for block (and crystalline) [23,26], copolymers synthesized in [bmim][BF_4_] have a tendency to form block copolymers due to the slower polymerization rate in this case. These copolymers are also probably more crystalline and thus are degraded slower than copolymers synthesized in [bmim][NTf_2_] (more randomness structure of the polymers). The microstructure of the copolymers also depends on the feed mole ratio of their monomers. At low *rac*-LA content, the degree of randomness remained low (0.17–0.28), which indicates the existence of block copolymers. When the feed mole ratio of *rac*-LA was high, there was a higher probability of occurrence of transesterification between lactidyl and caproyl units. These results are in a sharp contrast to those previously reported by Wei et al. [33] and Wahlberg et al. [25] for copolymerization of l-LA (or d,l-LA) and CL. They concluded much higher reactivity of LA than CL. Moreover, higher feed mole ratio of CL allows for transesterification. However, in our experiments provided in ILs, CL was the most active monomer, so the presence of the transesterification process depends on the existence of low active *rac*-LA.

## 3. Materials and Methods

### 3.1. Materials

*rac*-Lactide (*rac*-LA, 3,6-dimethyl-1,4-dioxane-2,5-dione, 99%) was purchased from Sigma-Aldrich Co., (Poznan, Poland) and further purified by crystallization from anhydrous toluene. ε-Caprolactone (CL, 2-oxepanone, 97%, Aldrich Co., Poznan, Poland) was dried and distilled over CaH_2_ at reduced pressure before using. Lipase B from *Candida* (*Pseudozyna*) *antarctica* (CALB) produced by submerged fermentation of a genetically modified *Aspergillus niger* microorganism and adsorbed on a macroporous resin (≥5000 U/g) were purchased from Sigma (Poznan, Poland). 1-butyl-3-methylimidazolium bis(trifluoromethylsulfonyl)imide, [bmim][NTf_2_] (≥98%, Aldrich Co., Poznan, Poland) and 1-butyl-3-methylimidazolium tetrafluoroborate, [bmim][BF_4_] (≥98%, Aldrich Co., Poznan, Poland) were used as received. Chloroform and toluene (Avantor, Gliwice, Poland) were used as received.

### 3.2. Synthesis of Copolymers of rac-Lactide and *ε*-Caprolactone

In a typical experiment, *rac*-LA was dissolved in ILs at temperature c.a. 130 °C in 10 mL glass ampoules under argon atmosphere. When the monomer was completely dissolved in ILs, a temperature was decreased to the reaction temperature (80 or 110 °C). Then, CL and CALB (10 wt. %) were introduced and the reaction could be proceeded for the desire time. At the end of the process, the enzyme was removed by filtration and the polymer’s products were isolated from ILs by extraction with toluene. After that toluene was removed by evaporation at room temperature under reduced pressure. Then, water-soluble monomers were washed away with the mixture of chloroform/distilled water and the polymers were dried in vacuo at room temperature for 4 days according to our previously reported method [12].

### 3.3. Spectroscopic Analysis of Polymers

The polymerization products were characterized by means of ^1^H- or ^13^C-NMR (using Varian 300 MHz recorded, Palo Alto, CA, USA) in deuterated chloroform (CDCl_3_) at room temperature. Tetramethylsilane was used as the internal standard.

A number-average molecular weight (*M_n_*) and their dispersity index (*M_w_*/*M_n_*) were determined by gel permeation chromatography (GPC). The GPC instrument (GPC Max + TDA 305, Viscotek) was equipped with Jordi DVB Mixed Bed columns (one guard and two analytical) at 30 °C in CH_2_Cl_2_ (HPLC grade, Sigma-Aldrich, MO, USA) and at a flow rate of 1 mL/min, with RI detection and calibration based on narrow PS standards (ReadyCal Set, Fluka).

## 4. Conclusions

The copolymers of *rac*-LA and CL were successfully obtained in the presence of a non-toxic catalytic system in ILs medium. Polymers without heavy metal residues in their structure and moderate molecular weight could be promising biomaterials, especially for drug delivery systems. We observed a higher conversion of *rac*-LA in a reaction in [bmim][NTf_2_] than in [bmim][BF_4_]. Moreover, the copolymers synthesized in [bmim][NTf_2_] with high *rac*-LA content have a more random structure, which indicates a more amorphous structure that can degrade faster. This demonstrates that [bmim][NTf_2_] can be preferably used as a medium for copolymerization of *rac*-LA or CL in comparison with [bmim][BF_4_] for biomedical applications.

## Figures and Tables

**Table 1:** Copolymerization of *rac*-LA and CL in the presence of CALB in ILs.

Entry	Solvent (1 mL)	Temp. (°C)	Time (days)	*f*_LA_^a^	*F*_LA_^b^	*rac-LA*conv. ^c^ (%)	Yield (%)	*M_n_* ^d^ (g/mol)	*M_w_*/*M_n_* ^e^	Average Dyad Relative Molar Fractions ^f^		
										LA-LA	LA-CL	CL-CL
A1	[bmim][BF_4_]	80	7	0.50	0.29	0.18	35			0.029	0.516	0.456
A2	[bmim][BF_4_]	80	14	0.50	0.27	0.75	43	1100	1.98	0.051	0.462	0.487
A3	[bmim][BF_4_]	110	14	0.50	0.31	0.69	45	2500	2.58	0.168	0.349	0.483
A4	[bmim][BF_4_]	80	7	0.33	0.25	0.65	42	700	1.54	0.023	0.464	0.513
A5	[bmim][BF_4_]	80	14	0.33	0.25	0.79	55	3100	3.82	0.028	0.343	0.630
A6	[bmim][BF_4_]	110	14	0.33	0.29	0.82	59	2800	3.10	0.108	0.352	0.540
A7	[bmim][BF_4_]	80	7	0.67	0.30	0.16	54			0.106	0.395	0.499
A8	[bmim][BF_4_]	80	14	0.67	0.28	0.18	56	1000	2.20	0.111	0.362	0.527
A9	[bmim][BF_4_]	110	14	0.67	0.42	0.65	57	2100	2.18	0.211	0.516	0.272
B1	[bmim][NTf_2_]	80	7	0.50	0.46	0.49	60			0.2212	0.479	0.300
B2	[bmim][NTf_2_]	80	14	0.50	0.46	0.86	63	1700	2.41	0.2698	0.391	0.340
B3	[bmim][NTf_2_]	110	14	0.50	0.44	0.90	69	2700	2.82	0.2247	0.432	0.344
B4	[bmim][NTf_2_]	80	7	0.33	0.31	0.92	55			0.0592	0.488	0.453
B5	[bmim][NTf_2_]	80	14	0.33	0.33	0.94	53	2400	1.80	0.1242	0.486	0.390
B6	[bmim][NTf_2_]	110	14	0.33	0.30	0.92	57	2900	3.28	0.1040	0.596	0.492
B7	[bmim][NTf_2_]	80	7	0.67	0.36	0.26	63			0.1119	0.498	0.390
B8	[bmim][NTf_2_]	80	14	0.67	0.62	0.59	52	3000	1.68	0.4438	0.375	0.181
B9	[bmim][NTf_2_]	110	14	0.67	0.60	0.92	61	1700	1.94	0.4000	0.412	0.188

a: feed mole fractions of *rac*-LA; b: mole fractions of lactidyl units in copolymers determined by ^1^H-NMR; c: determined by ^1^H-NMR; d: *M_n_* determined by GPC; e: *M_w_*/*M_n_* calculated from GPC; f: determined by ^1^H-NMR according to [23].

**Table 2:** Chain microstructure of obtained copolymers.

Sample	Microstructural Magnitudes of the Copolymers			
	$l_{LL}^{e}$	$l_{Cap}^{e}$	*R*	T_II_
A2	1.96	7.41	0.50	0.08
A3	1.52	12.88	0.25	0
A5	2.25	23.56	0.17	0.03
A6	1.23	16.14	0.21	0
A8	2.87	4.44	0.80	0
A9	-	-	-	0
B2	2.04	5.67	0.38	0.04
B3	3.52	5.53	0.41	0
B5	2.20	10.66	0.28	0.01
B6	2.08	14.38	0.23	0
B8	6.47	2.66	0.99	0
B9	5.81	2.48	0.67	0

$l_{LL}^{e}$: experimental average length of lactidyl blocks; $l_{Cap}^{e}$: experimental average length of caproyl blocks; *R*: degree of randomness; T_II_: yield of the second mode of transesterification [17,21].

## Supplementary Materials

The following are available online, Figure S1: Thermogravimetric analysis of ILs.

## References

[1] Nair L.S., Laurencin C.T. (2007). Biodegradable polymers as biomaterials. Prog. Polym. Sci..

[2] Seyednejad H., Ghassemi A.H., van Nostrum C.F., Vermonden T., Hennink W.E. (2011). Functional aliphatic polyesters for biomedical and pharmaceutical applications. J. Controll. Release.

[3] Labet M., Thielemans W. (2009). Synthesis of polycaprolactone: A review. Chem. Soc. Rev..

[4] Bergsma J.E., de Bruijn W., Rozema F., Bos R., Boering G. (1995). Late degradation tissue response to poly (l-lactide) bone plates and screws. Biomaterials.

[5] Oledzka E., Horeglad P., Gruszczyńska Z., Plichta A., Nałęcz-Jawecki G., Sobczak M. (2014). Polylactide conjugates of camptothecin with different drug release abilities. Molecules.

[6] Sobczak M., Kolodziejski W. (2009). Polymerization of cyclic esters initiated by carnitine and tin (II) octoate. Molecules.

[7] Kobayashi S., Shoda S.-I., Uyama H. (1995). Polymer Synthesis/Polymer Engineering.

[8] Albertsson A.-C., Srivastava R.K. (2008). Recent developments in enzyme-catalyzed ring-opening polymerization. Adv. Drug Deliv. Rev..

[9] Varma I.K., Albertsson A.-C., Rajkhowa R., Srivastava R.K. (2005). Enzyme catalyzed synthesis of polyesters. Prog. Polym. Sci..

[10] Sobczak M. (2012). Enzyme-catalyzed ring-opening polymerization of cyclic esters in the presence of poly(ethylene glycol). J. Appl. Polym. Sci..

[11] Sobczak M., Kamysz W., Tyszkiewicz W., Dębek C., Kozłowski R., Olędzka E., Piotrowska U., Nałęcz-Jawecki G., Plichta A., Grzywacz D. (2014). Biodegradable macromolecular conjugates of citropin: Synthesis, characterization and in vitro efficiency study. React. Funct. Polym..

[12] Piotrowska U., Sobczak M., Oledzka E., Combes C. (2016). Effect of ionic liquids on the structural, thermal, and in vitro degradation properties of poly(ε-caprolactone) synthesized in the presence of *Candida antarctica* lipase B. J. Appl. Polym. Sci..

[13] Domenek S., Ducruet V. (2016). Biodegradable and Biobased Polymers for Environmental and Biomedical Applications.

[14] Matsumura S., Mabuchi K., Toshima K. (1997). Lipase-catalyzed ring-opening polymerization of lactide. Macromol. Rapid Commun..

[15] Hans M., Keul H., Moeller M. (2009). Ring-opening polymerization of DD-Lactide catalyzed by novozyme 435. Macromol. Biosci..

[16] Düşkünkorur H.Ö., Bégué A., Pollet E., Phalip V., Güvenilir Y., Avérous L. (2015). Enzymatic ring-opening (co)polymerization of lactide stereoisomers catalyzed by lipases. Toward the in situ synthesis of organic/inorganic nanohybrids. J. Mol. Catal. B Enzym..

[17] Piotrowska U., Sobczak M. (2014). Enzymatic polymerization of cyclic monomers in ionic liquids as a prospective synthesis method for polyesters used in drug delivery systems. Molecules.

[18] Uyama H., Takamoto T., Kobayashi S. (2002). Enzymatic synthesis of polyesters in ionic liquids. Polym. J..

[19] Yoshizawa-Fujita M., Saito C., Takeoka Y., Rikukawa M. (2008). Lipase-catalyzed polymerization of l-lactide in ionic liquids. Polym. Adv. Technol..

[20] Chanfreau S., Mena M., Porras-Domínguez J.R., Ramírez-Gilly M., Gimeno M., Roquero P., Tecante A., Bárzana E. (2010). Enzymatic synthesis of poly-l-lactide and poly-l-lactide-*co*-glycolide in an ionic liquid. Bioproc. Biosyst. Eng..

[21] Mena M., López-Luna A., Shirai K., Tecante A., Gimeno M., Bárzana E. (2013). Lipase-catalyzed synthesis of hyperbranched poly-l-lactide in an ionic liquid. Bioproc. Biosyst. Eng..

[22] Mei Y., Kumar A., Gross R.A. (2002). Probing water-temperature relationships for lipase-catalyzed lactone ring-opening polymerizations. Macromolecules.

[23] Fernández J., Etxeberria A., Sarasua J.-R. (2012). Synthesis, structure and properties of poly (l-lactide-*co*-ε-caprolactone) statistical copolymers. J. Mech. Behav. Biomed..

[24] Dakshinamoorthy D., Peruch F. (2012). Block and random copolymerization of ε-caprolactone, l-, and *rac*-lactide using titanium complex derived from aminodiol ligand. J. Polym. Sci. A2.

[25] Wahlberg J., Persson P.V., Olsson T., Hedenström E., Iversen T. (2003). Structural characterization of a lipase-catalyzed copolymerization of ε-caprolactone and d, l-lactide. Biomacromolecules.

[26] Nalampang K., Molloy R., Punyodom W. (2007). Synthesis and characterization of poly (l-lactide-*co*-ε-caprolactone) copolymers: Influence of sequential monomer addition on chain microstructure. Polym. Adv. Technol..

[27] Kricheldorf H.R., Kreiser I. (1987). Polylactones. 13. Transesterification of Poly (l-Lactide) with Poly (Glycoude), Poly (β-Propio-Lactone), and Poly (ε-Caprolactone). J. Macromol. Sci. Chem..

[28] Kasperczyk J., Bero M. (1993). Coordination polymerization of lactides, 4. The role of transesterification in the copolymerization of L, L-lactide and ε-caprolactone. Macromol. Chem. Physic..

[29] Kricheldorf H.R., Boettcher C., Tönnes K.-U. (1992). Polylactones: 23. Polymerization of racemic and mesod, l-lactide with various organotin catalysts—Stereochemical aspects. Polymer.

[30] Bero M., Adamus G., Kasperczyk J., Janeczek H. (1993). Synthesis of block copolymers of ε-caprolactone and lactide in the presence of lithium t-butoxide. Polym. Bull..

[31] Zhou X., Hong L. (2013). Controlled ring-opening polymerization of cyclic esters with phosphoric acid as catalysts. Colloid Polym. Sci..

[32] Bero M., Kasperczyk J., Adamus G. (1993). Coordination polymerization of lactides, 3. Copolymerization of L, l-lactide and ε-caprolactone in the presence of initiators containing Zn and Al. Macromol. Chem. Phys..

[33] Wei Z., Liu L., Qu C., Qi M. (2009). Microstructure analysis and thermal properties of l-lactide/ε-caprolactone copolymers obtained with magnesium octoate. Polymer.

