# Supplementary material for: Characterization of Aliphatic Polyesters Synthesized via Enzymatic Ring-Opening Polymerization in Ionic Liquids
Source: Molecules. 2017 Jun 2;22(6):923. doi: 10.3390/molecules22060923 (PMC6152688; doi:10.3390/molecules22060923)
Supplement: Supplementary file 1 [file molecules-22-00923-s001.pdf]

Supplementary material

Article

Characterization of Aliphatic Polyesters Synthesized *via* Enzymatic Ring-Opening Polymerization in Ionic Liquids

Urszula Piotrowska [1,2,*], Marcin Sobczak [1,2] and Ewa Oledzka[1]

[1] Department of Biomaterials Chemistry, Chair of Inorganic and Analytical Chemistry, Faculty of Pharmacy with the Laboratory Medicine Division, Medical University of Warsaw, 1 Banacha St., Warsaw 02-097, Poland; marcin.sobczak@wp.pl (M.S.); eoledzka@wum.edu.pl (E.O.)

[2] Department of Organic Chemistry and Biochemistry, Faculty of Materials Science and Design, Kazimierz Pulaski University of Technology and Humanities in Radom, 27 Chrobrego St., Radom 26-600, Poland

* Correspondence: upiotrowska@wum.edu.pl or piotrowska_urszula@wp.pl ; Tel.: +48-22-572-07-55 (U.P.)

Materials and methods

Thermal characterization of ionic liquids (ILs).

Thermal properties of the ionic liquids were analyzed by thermogravimetric analysis (TGA) on TA Instruments Q50 (New Castle, DE USA). Samples (*ca.* 15 mg weight) were heated from room temperature to 700 °C at 10 °C·min^{-1} under nitrogen flow with a purge rate of 60 mL·min^{-1}.

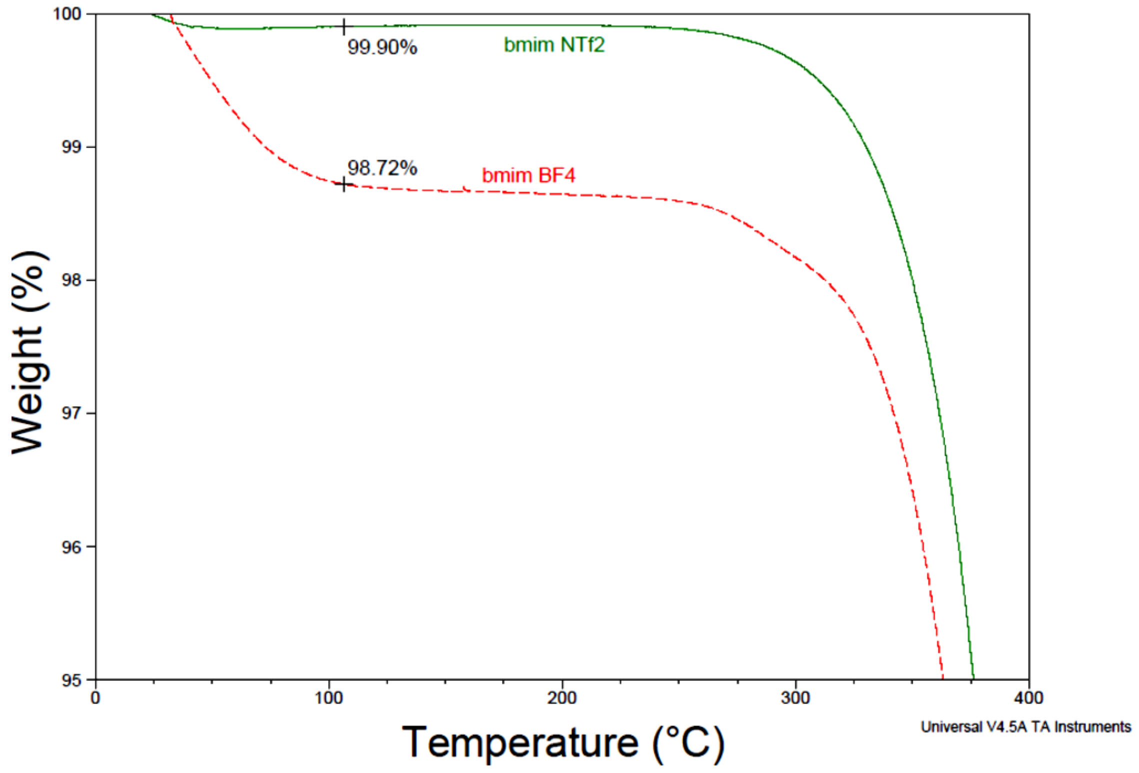

Figure S1 Thermogravimetric analysis of ILs.
